# Protective immunity by an engineered DNA vaccine for Mayaro virus

**DOI:** 10.1371/journal.pntd.0007042

**Published:** 2019-02-07

**Authors:** Hyeree Choi, Sagar B. Kudchodkar, Emma L. Reuschel, Kanika Asija, Piyush Borole, Michelle Ho, Krzysztof Wojtak, Charles Reed, Stephanie Ramos, Nathen E. Bopp, Patricia V. Aguilar, Scott C. Weaver, J. Joseph Kim, Laurent Humeau, Pablo Tebas, David B. Weiner, Kar Muthumani

**Affiliations:** 1 Vaccine & Immunotherapy Center, The Wistar Institute, Philadelphia, PA, United States of America; 2 Inovio Pharmaceuticals, Plymouth Meeting, PA, United States of America; 3 Department of Pathology, University of Texas Medical Branch, Galveston, TX, United States of America; 4 Institute for Human Infection and Immunity, University of Texas Medical Branch, Galveston, TX, United States of America; 5 Center for Tropical Diseases, University of Texas Medical Branch, Galveston, TX, United States of America; 6 Department of Microbiology and Immunology, University of Texas Medical Branch, Galveston, TX, United States of America; 7 Division of Infectious Diseases, Perelman School of Medicine, University of Pennsylvania, Philadelphia, PA, United States of America; Colorado State University, UNITED STATES

## Abstract

Mayaro virus (MAYV) of the genus alphavirus is a mosquito-transmitted emerging infectious disease that causes an acute febrile illness, rash, headaches, and nausea that may turn into incapacitating, persistent arthralgias in some victims. Since its discovery in Trinidad in 1954, cases of MAYV infection have largely been confined there and to the northern countries of South America, but recently, MAYV cases have been reported in some island nations in the Caribbean Sea. Accompanying these reports is evidence that new vectors, including *Aedes spp*. mosquitos, recently implicated in the global spread of Zika and chikungunya viruses, are competent for MAYV transmission, which, if true, could facilitate the spread of MAYV beyond its current range. Despite its status as an emerging virus, there are no licensed vaccines to prevent MAYV infection nor therapeutics to treat it. Here, we describe the development and testing of a novel DNA vaccine, scMAYV-E, that encodes a synthetically-designed consensus MAYV envelope sequence. *In vivo* electroporation-enhanced immunization of mice with this vaccine induced potent humoral responses including neutralizing antibodies as well as robust T-cell responses to multiple epitopes in the MAYV envelope. Importantly, these scMAYV-E-induced immune responses protected susceptible mice from morbidity and mortality following a MAYV challenge.

## Author summary

Mayaro virus (MAYV) is a mosquito-transmitted virus that causes fever, headache, chills, nausea and joint pain that can last for months after infection. The rising number of cases, due to increased mosquito circulation, and the threat of an epidemic emphasize its importance as an emerging virus, but there are no licensed vaccines to prevent Mayaro infection nor therapeutics to treat it. In this study, we gathered fundamental knowledge on how the immune system responds to MAYV infection, and we evaluated the efficacy of a novel, synthetic DNA envelope vaccine (scMAYV-E) in mice. Analysis of immune responses in mice demonstrated that this vaccine induces potent T cell immunity and antibodies. Mice who receive the vaccine and then are challenged with live MAYV are protected against Mayaro disease. This data provides evidence that the DNA-based MAYV vaccine may be able to prevent Mayaro disease. Thus, the scMAYV-E vaccine is a promising step forward for MAYV vaccine development. Future testing will evaluate whether this vaccine is a viable means to halt the spread of MAYV and protect individuals from Mayaro disease.

## Introduction

Mayaro virus (MAYV) is an alphavirus in the *Togaviridae* family originally identified on the island of Trinidad in 1954. MAYV infection can result in an acute febrile illness lasting 3 to 5 days with symptoms including rash, headache, nausea, vomiting, and diarrhea. Similar to chikungunya virus (CHIKV) infection, approximately 50% of MAYV-infected individuals develop painful recurrent arthralgia that can last for months after acute illness has cleared. Since its discovery, only sporadic cases of MAYV infection have been reported, mostly in tropical areas of South America [1, 2]. Serosurveys suggest that it may also be circulating in Central American countries [1, 3]. In 2015, the first case of MAYV infection outside of South America was reported on the island of Haiti, highlighting the potential for an expansion of the MAYV range to include island nations of the Caribbean Sea [2].

Alphaviruses are arthropod-borne viruses (arboviruses) transmitted between animal reservoirs and hosts via mosquitoes and other vectors. The primary vectors for MAYV are *Haemagogus spp*. mosquitos, which primarily reside in rural regions [2, 4, 5]. Most cases of MAYV infection have been in individuals that have entered forest environments due to work or travel. Recently, vector competence studies have shown that *Aedes aegypti* mosquitos have the capacity to transmit MAYV, sparking fears that the virus may spread beyond current endemic regions to possibly worldwide given the wider geographical distribution of *Aedes aegypti* [2, 6, 7]. In recent years, *Aedes spp*. mosquitos have been responsible for causing worldwide outbreaks of flaviviruses, including dengue and Zika [8–10]. With higher than expected seroprevalence in Central America [1,3, 11], it is evident that human infection with MAYV is becoming more common and widespread. As of currently, there are no approved vaccine or therapeutics available for blocking or treating MAYV infection, and current containment measures have focused on minimizing human-mosquito contacts through vector controls.

Alphaviruses like MAYV have single-stranded, positive sense RNA genomes that encode four nonstructural proteins (nsP1-4) and structural polyproteins; capsid, E3, E2, 6K and E1[12]. Along with E2 and E1, the envelope mRNA also encodes for a 6K polypeptide, which contributes to the processing and membrane insertion of E1 and precursor E2 (pE2) viral envelope glycoproteins that are cleaved during translation and get incorporated into mature virions [13, 14]. Occasionally, a frameshift during translation can lead to production of a transframe (TF) polypeptide that consists of the 6K polypeptide with additional amino acids on its C terminus [15]. Both 6K and TF appear to be involved in efficient virus budding [12, 16]. The E2 and E1 glycoproteins form heterodimers shortly after translation, and these heterodimers associate into trimers when virions assemble. The E2 glycoprotein is primarily involved in virus attachment to host cells while the E1 protein mediates the fusion of the virus and host cell. Both E1 and E2 on the surface of virions are targets of anti-MAYV antibody responses [12, 17, 18].

A protective vaccine targeting MAYV would be an important tool for impeding its spread as well as reducing or eliminating disease caused after infection. To date, two MAYV vaccines have been developed and shown to be immunogenic in mice models: (1) an inactivated virus vaccine [19] and (2) a live-attenuated virus vaccine [20]. Here, we describe the development of a novel synthetic DNA-based vaccine targeting MAYV. Our lab has previously used this platform to develop potent vaccines against diverse infectious agents, including HIV-1, Ebola virus, Middle East respiratory syndrome coronavirus (MERS-CoV), and Zika virus (ZIKV) [21–23]. DNA-based vaccines are cheaper to design, manufacture, and deploy than conventional vaccine platforms, and they are capable of inducing both humoral and cellular responses with virtually no risk of causing disease themselves [21, 22, 24]. Importantly, DNA vectors are non-immunogenic, thus there is no reduction in potency after multiple administrations. While first generation DNA vaccines exhibited poor and inconsistent immunity, improvements in plasmid and antigen designs combined with the addition of electroporation (EP)-enhanced vaccine delivery have greatly improved the immunogenicity of these vaccines. A recent phase I clinical trial of a novel synthetic ZIKV vaccine, GLS-5700, found that it induced both cellular and humoral responses, including neutralizing antibodies, in the vast majority of study volunteers, and passive transfer of post-vaccination sera from volunteers completely protected mice from morbidity and mortality following ZIKV challenge [22]. A phase II clinical trial of a therapeutic DNA vaccine (VGX-3100) encoding consensus sequences of human papilloma virus (HPV) E6 and E7 proteins induced antigen-specific humoral and cellular responses in volunteers after EP-enhanced delivery, and these responses could mediate viral clearance and clinical regression of CIN2/3 cervical dysplasia in volunteers [24].

The vaccine described here, scMAYV-E, encodes a synthetically designed, consensus full-length MAYV envelope antigen sequence. EP-enhanced delivery of scMAYV-E into immunocompetent mice induced high levels of cellular responses to multiple MAYV-E epitopes along with robust antibody responses that could neutralize MAYV infection *in vitro*. Immunization of interferon α/β receptor knockout mice (IFNAR^-/-^; A129) with scMAYV-E protected the mice from morbidity and mortality following MAYV challenge, where the protection in this model was primarily due to vaccine-induced humoral responses. The robust immunogenicity of the scMAYV-E vaccine demonstrated here supports the need for further testing of this vaccine as a viable means to halt the spread of this virus and protect individuals from MAYV disease.

## Materials and methods

### Cell culture

Human embryonic kidney 293T (HEK293T; ATCC-CLR-N268) and Vero CCL-81 (ATCC #CCL-81) (ATCC, Manassas, VA, USA) cells were cultured in D10 media: Dulbecco Modified Eagle's Medium (Invitrogen Life Science Technologies, San Diego, CA, USA) supplemented with 10% heat-inactivated fetal calf serum (FCS), 3 mM glutamine, 100 U/ml penicillin, and 100 U/ml streptomycin [23]. Mouse splenocytes were cultured in R10 media: (RPMI1640, Invitrogen Life Science Technologies, San Diego, CA, USA) supplemented with 10% heat-inactivated FCS, 3 mM glutamine, 100 U/ml penicillin, and 100 U/ml streptomycin. All cell types were cultured in incubators set to 37°C and 5% CO_2_.

### MAYV vaccine construction and expression

The synthetic MAYV vaccine DNA construct encodes a full-length MAYV envelope sequence. The consensus gene insert was computationally optimized for improved expression. The construct was synthesized commercially (Genscript, NJ, USA) and then sub-cloned into a modified pVax1 vaccine expression vector under the control of the cytomegalovirus immediate-early promoter as described previously [23].

HEK293T cells were plated in six-well plates at 6x10^5^ cells/well and transfected 24 hours later with scMAYV-E and pVax1 empty vector control plasmids using GeneJammer transfection reagent (Agilent Technologies, Santa Clara, CA, USA) according to the manufacturer's instructions. The transfection was carried out in Opti-MEM medium (Invitrogen). The transfected supernatants and cell lysates were collected 48 hours post transfection, and antigen expression was confirmed by western blot analysis. Cells were washed with phosphate-buffered saline (PBS) and lysed with lysis buffer containing 50 mM HCl, 150 mM NaCl, 1% Nonidet P-40, 1% Triton X-100, 0.1% sodium dodecyl sulfate, and a cocktail of protease inhibitors (Roche, Basel, Switzerland) on ice for 30 minutes with intermediate vortexing. After 10 minutes of centrifugation at 13,000 rpm, the supernatant was collected and analyzed by sodium dodecyl sulfate-12% polyacrylamide gel electrophoresis and transferred to a nitrocellulose membrane for immunoblotting with antisera (1:100 dilution) against scMAYV-E. Secondary antibodies coupled to horseradish peroxidase (HRP) were used at a dilution of 1:5,000. Next, the membrane was stripped using NewBlot Nitrocellulose 5x Stripping Buffer (Li-Cor, Nebraska, USA) then probed with β-actin rabbit monoclonal antibody (1:1,000) (Li-Cor, USA) as a loading control. For Immunofluorescence analysis, cells were seeded on top of coverslips in a 6-well cell culture plate. After washing three times with PBS, the cells were incubated for an hour at 37°C with a Fluorescein isothiocyanate (FITC)-conjugated goat anti-human IgG (Santa Cruz Biotechnology Inc., USA). The nucleus was stained with 4′, 6-diamidino-2-phenylindole (DAPI) at room temperature for 20 minutes. PBS washes were performed after each incubation step. The samples were subsequently mounted onto glass slides using Fluoroshield Mounting Medium (Abcam, USA) and were viewed under a confocal microscope (LSM710; Carl Zeiss). The resulting images were analyzed using the Zen software [21, 25].

### Animals and DNA immunizations with electroporation

Five- to eight-week old female C57BL/6 mice (The Jackson Laboratory, Bar Harbor, ME, USA) were housed and vaccinated in a light-cycled, temperature- and humidity-controlled animal facility. Four- to six-week old mice of C57BL/6 background deficient in the interferon-α/β receptors (IFNAR^-/**-**^; A129) were purchased from The Jackson Laboratory (MMRRC Repository-The Jackson Laboratory, USA) and established a breeding colony approved by the Wistar Institutional Animal Care and Use Committee (IACUC# 112842X_0). Animals were bred and housed in a barrier animal facility at the Wistar Institute. All murine studies were performed in accordance with the recommendations from the National Institutes of Health (NIH) and the Wistar Institute Institutional Animal Care and Use Committee.

For DNA immunization, five- to eight-week-old female C57BL/6 mice and four- to six-week old IFNAR^**-/-**^ mice of mixed sex were delivered 25 μg of DNA in a total volume of 30 μl of sterile water by a syringe into the anterior tibialis (TA) muscle. The same site is immediately electroporated by the CELLECTRA adaptive constant current enhanced electroporation (EP) delivery device (Inovio Pharmaceuticals, PA, USA), where a three-pronged minimally invasive device is inserted 2 mm into the TA muscle. Each prong consists of 26-gauge, solid stainless-steel electrode, and triangulated square-wave pulses of 0.1 Amps are delivered at 52 msec/pulse twice with a 1 second delay at the insertion site. Further details of the EP usage have been previously described in detail [26, 27]. Blood was collected by the submandibular method preceding the DNA injection and EP procedure. All mice were anesthetized with 2–5% isoflurane (Phoenix, Clipper, MO, USA) during procedures. Each group received one, two, or three immunizations at 2-week intervals, and mice were euthanized one week following the last immunization.

### Splenocyte isolation and IFN-γ ELISpot assay

Spleens were dissected and individually crushed with the use of a Stomacher device (Seward, UK). Splenocytes were strained with a 40 μm cell strainer (ThermoFisher, USA) and treated 5 minutes with Ammonium-Chloride-Potassium (ACK) lysis buffer (Quality Biologicals, MD, USA) to lyse red blood cells. The splenocytes were resuspended in R10 and used in the Mouse IFN-γ ELISpot PLUS assay (Mabtech, USA) according to the manufacturer’s instructions. Briefly, 2x10^5^ splenocytes from the scMAYV-E or pVax1 control immunized mice were added to each well and incubated for 18 hours at 37°C in 5% CO_2_, either in the presence of media alone (negative control), media with Cell Activation Cocktail (BioLegend, USA) containing pre-mixed phorbol 12-myristate-13-acetate (PMA) and ionomycin (positive control), or media with peptide pools (1 μg/ml) consisting of linearly pooled 20 individual peptides that are 15-mers overlapping by 9 amino acids spanning the length of the MAYV envelope protein. Spots were formed by the addition of 5-bromo-4-chloro-3-indolyl-phosphate/nitro blue tetrazolium (BCIP/NBT) color development substrate (R&D Systems, USA). Spot forming units (SFU) were quantified by an automated ELISpot reader (CTL Limited, USA). The average number of SFU from the media alone wells was subtracted from each stimulated well, and the data was adjusted to SFU per 10^6^ splenocytes [22].

### ELISA for detection of Ag-specific antibody

MaxiSorp high-binding 96-well ELISA plates (ThermoFisher, USA) were coated with commercial recombinant MAYV E1 Envelop Glycoprotein (Alpha Diagnostic, San Antonio, TX, USA; MAYV11-R-100) and MAYV E2 Envelop Glycoprotein (Alpha Diagnostic, USA; MAYV21-R-100) at a concentration of 0.5 μg/mL in coating buffer (0.012 M Na_2_CO_3_, 0.038 M NaHCO_3_, pH 9.6) at 4°C overnight. Plates were washed 5 times with PBS buffer solution containing 0.01% Tween-20 (PBST) (ThermoFisher, USA), and blocked with 10% FBS in PBS at 37°C for 1 hour. Serum samples were serially diluted (starting 1:50, dilution factor 3.16) in PBS containing 1% FBS, and 100 μl was added to each well. After incubation at 37°C for 2 hours, the plates were washed 5 times with PBST and then incubated with HRP-labeled goat anti-mouse IgG (Sigma-Aldrich, USA), at 37°C for 1 hour. After the final wash, 100 μl of fresh 3,3’5,5’-Tetramethylbenzidine (TMB) Substrate (Sigma-Aldrich) was added per well and incubated for 10 minutes. The reaction was stopped by adding 50 μl of 2 M H_2_SO_4_, and the optical density of the plate was measured at 450 nm by Biotek ELISA plate reader (Biotek, USA). The antibody endpoint titer was defined as the highest dilution of a serum sample with OD values > (mean + 3SD) of pVax1 vaccinated mice. Samples with a titer <50 were given an endpoint titer of 1. All assays were done in triplicate. For mouse IgG subtyping, Pierce Rapid Antibody Isotyping Kit (ThermoFisher, USA) was used with 1:100 dilution of pVax1 mouse sera or scMAYV-E immune sera from C57BL/6 background.

### Flow cytometry and intracellular cytokine staining assay

2x10^6^ single-cell suspended mouse splenocytes were added per well to a U-bottom 96-well plate (ThermoFisher). Cells were stimulated for 5 hours at 37°C in 5% CO_2_, either in the presence of media alone (negative control), media with Cell Activation Cocktail (BioLegend) containing pre-mixed PMA and ionomycin (positive control), or media with MAYV envelope peptides (1μg/ml) spanning the length of the entire protein, where all of the samples contained a protein transport inhibitor cocktail (eBioscience, San Diego, CA, USA). Upon completed stimulation, the cells are washed with FACS buffer (PBS containing 0.1% sodium azide and 1% FBS). Cells were stained for the surface proteins using fluorochrome-conjugated antibodies per the manufacturer’s instructions (BD Biosciences, San Diego, CA, USA). The cells were washed again with FACS buffer, then fixed and permeabilized using BD Cytofix/Cytoperm (BD Biosciences) per the manufacturer’s protocol before the intracellular cytokines were stained using fluorchrome-conjugated antibodies (BD Biosciences). The following antibodies were used for surface staining: LIVE/DEAD Fixable Violet Dead Cell stain kit (Invitrogen); CD19 (V450; clone 1D3; BD Biosciences); CD4 (FITC; clone RM4-5; eBioscience); CD8α (APC-Cy7; clone 53–6.7; BD Biosciences); CD44 (A700; clone IM7; BioLegend). For intracellular staining the following antibodies were used: IFN-γ (APC; clone XMG1.2; Biolegend); TNF-α (PE; clone MP6-XT22; eBioscience); CD3ε (PerCP/Cy5.5; clone 145-2C11; Biolegend); IL-2 (PeCy7; clone JES6-SH4; eBioscience). The LSRII flow cytometer was outfitted with the following lasers and bandpass filters: (i) violet (405 nm)– 450/50, 525/50, 560/40, 585/42, 605/40, 660/40, 705/70, 780/60; (ii) blue (488 nm)– 530/30, 695/40; (iii) green (532 nm)– 575/25, 610/20, 660/20, 710/50, 780/60; and (iv) red (640nm)– 670/30, 710/50, 780/60. All data was collected using an LSRII flow cytometer (BD Biosciences) and analyzed using FlowJo software (Tree Star, Ashland, OR, USA) and SPICE v5. Boolean gating was performed using FlowJo software to examine the polyfunctionality of the T cells from vaccinated animals [21, 23, 24].

### Mayaro viral challenge experiments in IFNAR^-/-^ mice

The Trinidad Regional Virus Laboratory (TRVL) 15537 strain of MAYV was obtained from ATCC (ATCC VR-1863), passaged once through Vero cell culture, and quantified as previously described [20, 28]. One week after the second immunization, 10 mice from either scMAYV-E vaccinated or pVax1 vaccinated groups were challenged with 10^2^ plaque-forming units (PFU) of MAYV diluted in 100 μl of sterile PBS delivered by a gradual intraperitoneal (i.p.) inoculation. Mice were weighed daily and evaluated for clinical signs of infection as follows: (1) decreased mobility; (2) hunched posture; (3) footpad swelling; (4) decreased grip strength of the hindlimb; (5) paralysis of hindlimb(s); (6) moribund. The Wistar Institute IACUC does not approve death as an endpoint. Mice were euthanized if (1) weight loss was sustained for 3 days or more and total weight loss reaches 20% of the body weight, (2) mice exhibited 3 or more signs of clinical symptoms as listed above concurrently for over 3 days, or (3) mice were moribund. All mice that exhibited signs and symptoms of MAYV infection lasted no more than 7 days during the experiment(s). Whole blood was collected on day 6 post challenge for Mayaro viral titer quantification as described previously [20, 28], and the footpad swelling of individual mouse was measured with a caliper on the same day. Eight days post challenge, the number of surviving mice was noted and humanely euthanized.

### Passive transfer of immune sera from immunized IFNAR^-/-^ to naive IFNAR^-/-^ mice

Four- to six-week old IFNAR^-/-^ mice were immunized twice at a two-week interval. One week after the second immunization, immune sera were isolated from whole blood and combined into a single pool per group. Passive transfer of immune sera was performed by intraperitoneal (i.p.) injection at 200 μl per mouse into four- to six-week old naive IFNAR^-/-^ mice. Mice receiving immune sera from the pVax1 immunized group or PBS served as negative controls. All groups were challenged with 10^2^ PFU of wild-type TRVL 15537 strain of MAYV and monitored daily as described above.

### Adoptive transfer of splenocytes from immunized IFNAR^-/-^ to naive IFNAR^-/-^ mice

Four- to six-week old IFNAR^-/-^ mice were immunized twice at a two-week interval. One week after the second immunization, all mice were euthanized, and spleens were collected and processed as single-cell suspensions. The cell viability was examined by Trypan Blue dye exclusion staining using a Countess II Automated Cell Counter (ThermoFisher). Adoptive transfer was performed by i.p. inoculation (2x10^6^ cells/200 μl) into four- to six-week old naive IFNAR^-/-^ mice. A group receiving splenocytes i.p. (200 μl) from the pVax1 immunized mice or PBS served as negative controls. All groups were challenged with 10^2^ PFU of wild-type TRVL 15537 strain of MAYV and monitored daily as described above.

### Neutralization assay

PRNT assay was carried out to detect and quantify the presence of neutralizing antibodies in the immunized mouse serum samples as previously described [25, 27, 29]. Heat-inactivated (56°C, 30 minutes) immune sera were diluted serially, and 150 μl of each diluted sample was mixed with an equal volume of 10^2^ PFU of wild-type TRVL 15537 strain of MAYV, followed by incubation at 37°C for 1.5 hour for a virus-antibody neutralization reaction. 100 μl of the virus and serum mixture was then used to inoculate a monolayer of Vero cells in a 12-well plate followed by an incubation at 37°C for 1.5 hour with rocking every 15 minutes. Next, the supernatant was removed from each well, and a layer of 2% methyl cellulose was added. After further incubation at 37°C with 5% CO_2_ for 3 days, the cells were fixed, stained with Crystal Violet (ThermoFisher), and plaque numbers were recorded as described [28]. MAYV alone without serum incubation served as negative control. After washing the stained cells with distilled water and air-drying the plates, the number of foci per well were counted using a stereomicroscope. The percentage of infectivity was calculated as: % reduction in infection = {1-(number of plaques from serum samples / number of plaques from negative control)} x100.

### Macrophage infection of MAYV and cell viability assay

Purified CD14^+^ human monocytes were obtained from the University of Pennsylvania Immunology Core (Philadelphia, USA). Human monocyte-derived macrophages (MDM; 1x10^6^/well) were cultured in a 6-well plate in Macrophage Base Medium DXF (PromoCell GmbH, Germany) supplemented with 60 ng/mL of granulocyte-macrophage colony-stimulating factor (GM-CSF) recombinant protein (R&D Systems, USA). The culture was incubated without disturbance at 37°C with 5% CO_2_ for 3 days. and MDMs were washed once with PBS prior to infection. Cells were infected with 10^2^ PFU of TRVL 15537 strain of MAYV that was preincubated for 1 hour at 37°C with either pVax1 immune sera or a 100-fold dilution of pooled day 35 immune sera from scMAYV-E vaccinated mice. The same media containing virus-pVax1 sera or virus-scMAYV-E sera mixtures were added to washed MDMs in a 6-well plate that were then kept for 1 hour at 37°C with a rocking interval of 15 minutes. The supernatant was removed following incubation, and Macrophage Base Medium DXF (PromoCell GmbH, Germany) was added to each well and further incubated at 37°C with 5% CO_2_ for 48 hours. Uninfected and infected macrophages were stained with Live Cell Labeling Kit- Green Fluorescence-Cytopainter (Abcam, Cambridge, MA, USA) according to the manufacturer’s instructions. Stained macrophages were imaged on a microscope (EVOS Cell Imaging Systems; Life Technologies) and % live cells (i.e., Labeling Dye Green^+^) were assessed by visual inspection of images from six different reviewers assessing the inhibited infection relative to base-line by 90% [22]. A monolayer of Vero CCL-81 cells plated on 12-well plates were inoculated with 200 μl of supernatants from MAYV-infected MDMs that were previously pre-incubated MAYV with either pVax1 or scMAYV-E sera. After 36 hours of incubation, viability of the Vero CCL-81 cells was examined by Trypan Blue dye exclusion staining using a Countess II Automated Cell Counter (ThermoFisher). The assays were done in triplicates, and each dot represents the cell viability from a single well +/- SEM. The experiment was repeated twice.

### Ethics statement

The Wistar Institute Animal Care and Use Committee (IACUC) approved the animal experiments under the protocol #112770 in accordance with the Guide for the Care and Use of Laboratory Animals by the National Research Council of the National Academies (8^th^ Edition, 2011). The Wistar Institute IACUC and the Animal Facility comply with all applicable federal statues and state regulatory provisions that include but are not limited to the following: the Animal Welfare Act of the U.S. Department of Agriculture (USDA), the U.S. Government Principles for the Utilization and Care of Vertebrate Animals Used in Testing, Research, and Training developed by the Interagency Research Animal Committee (IRAC) and the Public Health Service (PHS) Policy on Humane Care and Use of Laboratory Animal of the National Institute of Health (NIH). Appropriate practices and procedures as defined in the Biosafety in microbiological and biomedical laboratories (US Dept. of Health and Human Services) were used in sample handling. Samples were stored at -80°C in a bio safety level-2 (BSL-2) facility at the Vaccine & Immunotherapy Center, The Wistar Institute, PA, USA.

### Statistical analysis

All results are representative of those from at least two independent experiments with similar results. Graphs, standard curves, and pie charts were made using GraphPad Prism (version 4.0) software. IC_50_ values were calculated using a non-linear regression of the reciprocal of the serum dilution compared to the control. The survival data for mouse experiments were graphed using Kaplan-Meier survival curves. Two-tailed *p* values were calculated by log-rank (Mantel-Cox) test for nonparametric data using GraphPad Prism (version 4.0) software.

## Results

### Synthetic consensus Mayaro DNA vaccine development and characterization

**Fig 1:**
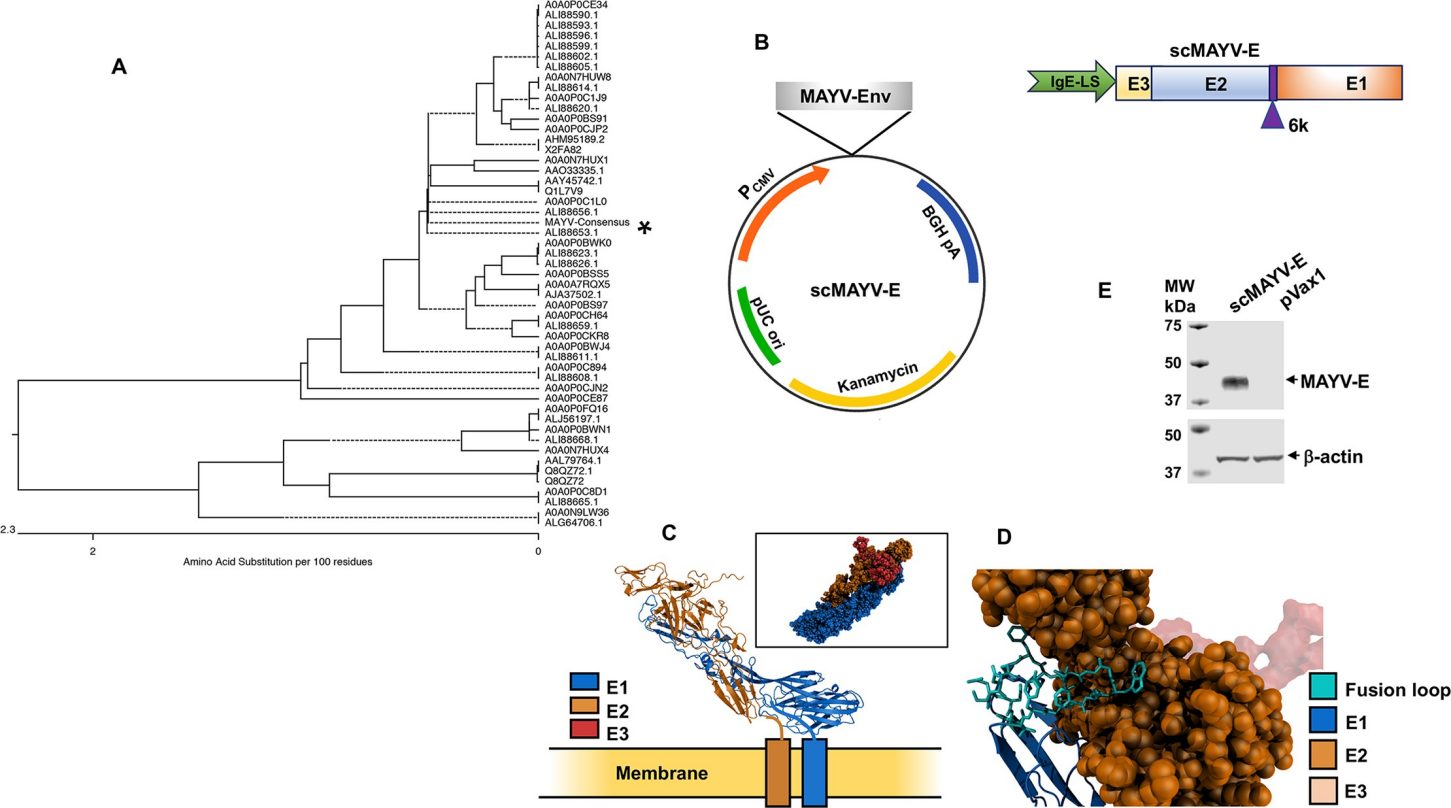
Development and characterization of a synthetic consensus Mayaro DNA vaccine. A) Phylogenic analysis based on neighbor joining evaluation of MAYV-envelope sequences deposited in GenBank. The position of the scMAYV-E vaccine sequence in this tree is noted with an asterisk ‘*’ (B) Schematic of the scMAYV-E vaccine construct generated. scMAYV-E encodes all three envelope glycoproteins (E1, E2, and E3) and the 6K/TF polypeptide linked by protease cleavage sites. The IgE-leader sequence was inserted at the 5’ end to increase protein expression. (C) A computer-generated MAYV antigen model. (D) View of E1 showing fusion loop in cyan. The fusion loop is generally conserved among the alphaviruses. E3 (transparent red) is shown to provide orientation. The highly conserved MWGG sequence is visible at the loop’s rightmost position. The M residue is buried and predicted to interact with nearby TYR and PRO residues of E2. The backbone portion of M participates in H-bonds with the nearby G at position i+3 to form a b-turn. (E) Western analyses of lysates from scMAYV-E or pVax1 transfected 293T cells incubated with pooled day 35 sera from scMAYV-E immunized mice. β-actin was used as a loading control for transfected 293T cell lysates.

We employed bioinformatics and synthetic DNA technologies to create a novel DNA vaccine encoding a full-length MAYV envelope gene sequence comprised of the E1, E2, and E3 glycoprotein domains as well as the 6K/TF polypeptides. The synthetic DNA insert was created by aligning full-length envelope genomic sequences from multiple MAYV strains deposited in the GenBank-NCBI (National Center for Biotechnology Information) database and determining the most common conserved amino acid at each position. Consensus antigen sequences account for genetic variability that occurs over time in a sequence and thus mapped at the phylogenetic midpoint (Fig 1A). Studies show that synthetic consensus sequences can focus immune responses against conserved sites as well as broaden T cell immunity [23, 30]. To improve the transcription and translation of the vaccine inserts, modifications to the insert sequences were made prior to cloning into the modified pVax1 vaccine expression vector namely the addition of an immunoglobulin E (IgE) leader sequence to the N-terminus (Fig 1B) along with codon and RNA optimization of the sequences [21]. Reference models of the scMAYV-E antigen made using Discovery Studio 4.5. software indicate that its predicted structure matches that of a wild-type MAYV envelope with the fusion loop at the end of domain E1 tucked into a fold in domain E2 (Fig 1C and 1D), thus preserving important envelope functional sites. Expression of the scMAYV-E antigen was confirmed *in vitro* through western analyses of 293T cell lysates transfected with scMAYV-E vaccine (Fig 1E).

### scMAYV-E induces binding and neutralizing antibody responses in mice

**Fig 2:**
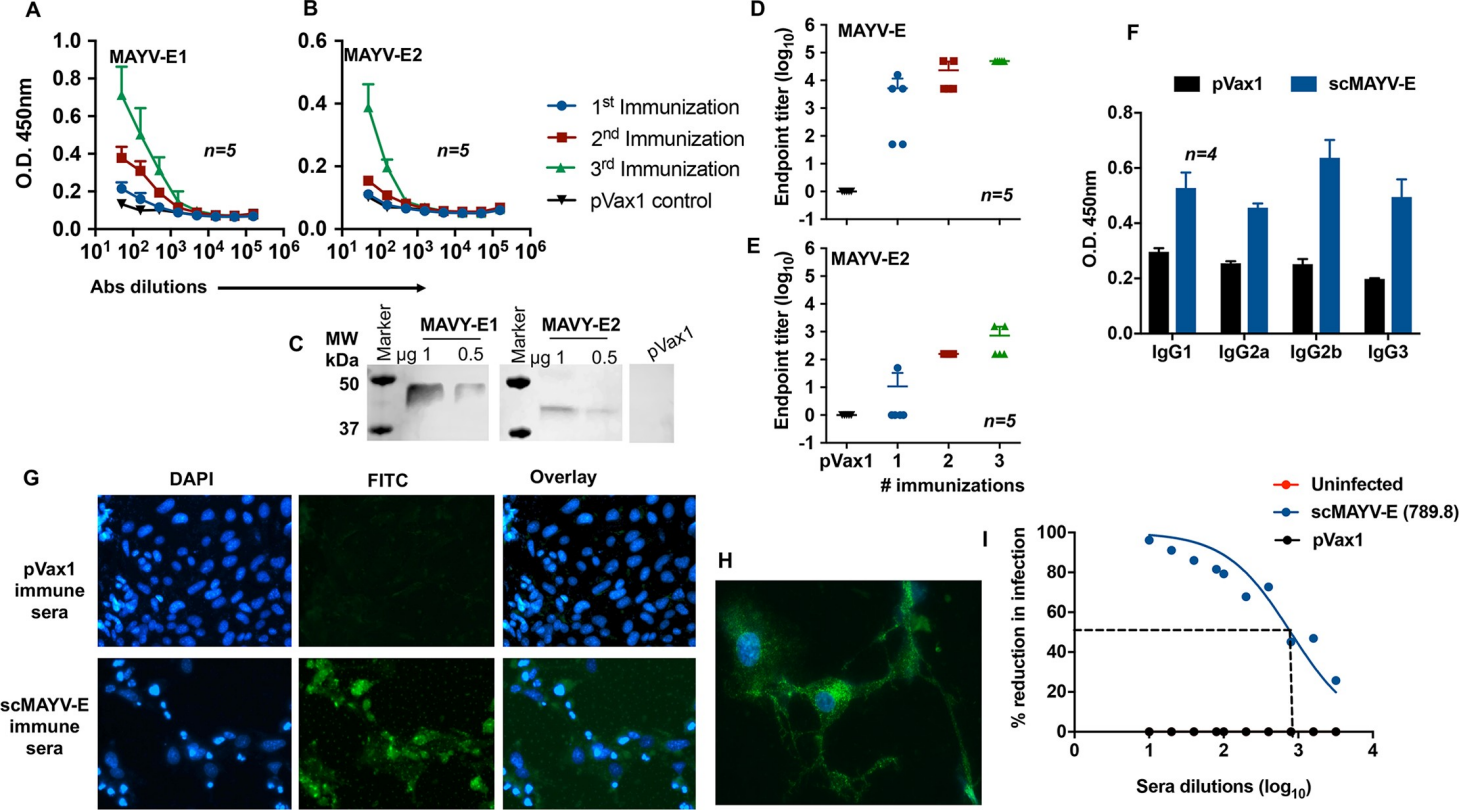
scMAYV-E vaccine induces a robust, MAYV-specific humoral response in mice including neutralizing antibodies. (A) ELISA of sera from scMAYV-E immunized mice. C57BL/6 mice (n = 5) were immunized three times using EP-enhanced i.m. injection with 25 μg of scMAYV-E or pVax1 empty vector plasmid at 2-week intervals with sera collected one week after each immunization. Half-log dilutions of sera from individual mice were evaluated for their binding capacity to a commercial recombinant MAYV-E1 (rE1) protein. (B) Binding ELISA of murine sera from (A) using a commercial recombinant MAYV-E2 (rE2) protein. (C) Western blot analysis of scMAYV-E immunized murine sera. Pooled day 35 sera from the aforementioned experiment was used as a primary antibody to probe rE1, rE2 glycoproteins and pVax1-transfected 293T cell lysates as a negative control. (D) rE1-specific IgG endpoint titer of scMAYV-E vaccinated mouse sera after each immunization. The antibody endpoint titer was defined as the highest dilution of a serum sample with OD values > (mean + 3SD) of pVax1 vaccinated mice. Samples with a titer <50 were given an endpoint titer of 1. (E) rE2-specific IgG endpoint titer of scMAYV-E vaccinated mouse after each immunization. Day 35 pVax1 sera was used to calculate the endpoint titer as performed in (D). (F) IgG subclass isotyping of C57BL/6 pVax1 mouse sera and scMAYV-E mouse sera one week post third immunization (day 35). IgG1, IgG2a, IgG2b, and IgG3 for both groups shown (n = 4). (G) Indirect immunofluorescence assay of MAYV-infected Vero cells incubated with pooled day 35 sera from pVax1 or scMAYV-E immunized mice followed by FITC-tagged anti-mouse IgG secondary antibody (green) and DAPI (blue) to identify nuclei. (H) Indirect immunofluorescence assay of MAYV-infected U87 neuronal cells incubated with pooled day 35 sera from scMAYV-E DNA immunized mice followed by FITC-tagged anti-mouse IgG secondary antibody (green) and DAPI (blue) to identify nuclei. (I) Plaque reduction neutralization assay (PRNT_50_) of heat-inactivated pooled day 35 sera from uninfected naive, pVax1, or scMAYV-E immunized mice. Serial two-fold dilutions of sera were incubated with 10^2^ PFU of MAYV for 1.5 hours and then added to wells of confluent Vero cells. Plaque formation in wells was scored at 3 days post infection and % reduction of plaque formation was calculated in comparison to plaques formed in wells receiving virus only. PRNT_50_ value is calculated by a non-linear regression analysis using PRISM software.

The immunogenicity of the scMAYV-E vaccine was evaluated in C57BL/6 mice. Initially, groups of mice were immunized three times, two weeks apart, with 25 μg of either scMAYV-E or an pVax1 empty vector plasmid using EP-enhanced intramuscular (i.m.) delivery [31]. Immunized mice were bled on day 0 and one week after each injection to obtain sera, which were assayed for the presence of antibodies to MAYV envelope using ELISA with commercially available MAYV E1 and E2 glycoproteins. The results show that all mice develop anti-MAYV E1 antibodies after a single immunization (Fig 2A), and the anti-MAYV E2 IgG response was boosted by both a second and third immunization (Fig 2B). The presence of anti-E1 and anti-E2 responses in murine sera post third immunization (day 35) was confirmed via western blot analysis using commercially available E1 and E2 glycoproteins as loading antigens and pVax1-transfected 293T cell lysates as a negative control (Fig 2C). Multiple immunizations also enhanced the affinity of the vaccine-induced anti-MAYV responses against E1 and E2 glycoproteins as evidenced by increasing endpoint titers after the second and third immunizations (Fig 2D and 2E). There is a comparable increase in total IgG1, IgG2a, IgG2b, and IgG3 subtypes after the third immunization (Fig 2F). Both Vero-CCL81 (Fig 2G) and U-87 MG neuronal cells (Fig 2H) infected with the wild-type MAYV could be identified by indirect immunofluorescence assay using pooled day 35 sera from scMAYV-E immunized mice but not when using pooled day 35 pVax1 sera.

Studies on related alphaviruses including CHIKV have established anti-viral antibodies as a primary correlate of protection [27, 32, 33]. We next evaluated whether the antibody response elicited by our scMAYV-E vaccine in mice could neutralize MAYV infection *in vitro*. A plaque reduction neutralization test (PRNT_50_) was performed on pooled day 35 sera from scMAYV-E immunized, pVax1 immunized, or uninfected control mice. Antibodies in scMAYV-E vaccinated mice neutralized MAYV infection of Vero-CCL81 cells with a high neutralizing titer (IC_50_ = 789.8) while pVax1 immune sera or uninfected control sera were not able to neutralize the virus at the highest serum dilution (1:10) (Fig 2I). These results indicate that scMAYV-E induces robust, MAYV-specific humoral responses in mice that are capable of blocking MAYV infection *in vitro*.

### scMAYV-E immune sera protect human macrophages from MAYV infection-induced cell death

**Fig 3:**
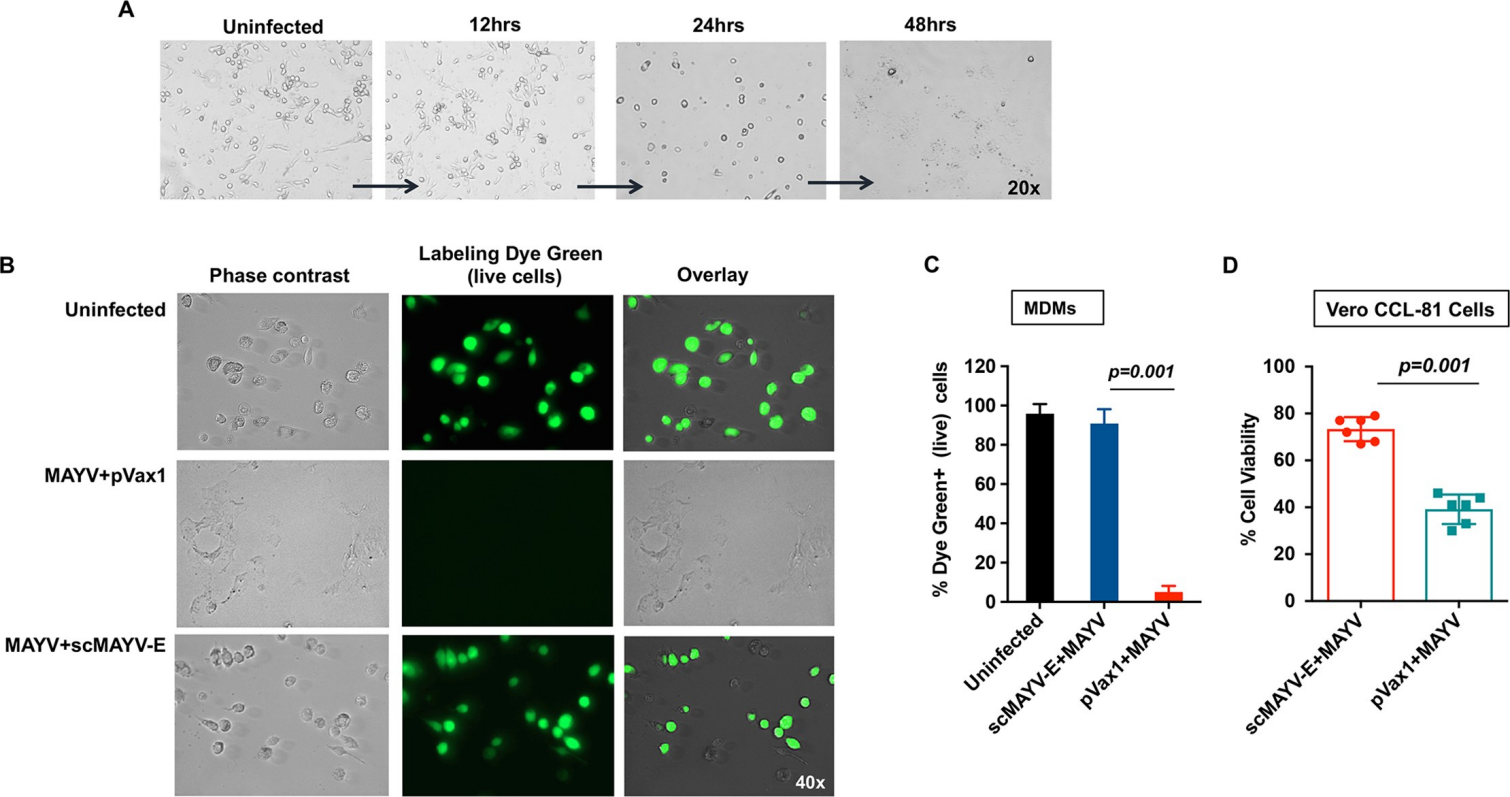
scMAYV-E immune sera protect monocyte derived macrophages (MDMs) from MAYV infection-induced cell death. (A) Phase contrast images of MAYV-infected MDMs over time. Magnification: x20. (B) Phase contrast and fluorescent images of MDMs infected with MAYV preincubated with immune sera. 3 day-old cultures of MDMs were treated with DMEM media only, MAYV plus pVax1 sera, or MAYV preincubated with 1:100 dilution of pooled day 35 (post third immunization) immune sera from scMAYV-E immunized mice. After 48 hours of co-culture, the cells were fixed, permeabilized, and stained with Live Cell Labeling Kit-Green Fluorescence-Cytopainter, which stains only live cells with Labelling Dye Green. Magnification: ×40. (C) Histogram comparing the percentage of live cells in the control and experimental groups evaluated by Labelling Dye Green signals from six independent evaluations of the infected MDMs in Fig 3B. (D) Percent viability of Vero CCL-81 cells inoculated 36 hours with supernatants from the infected MDM cultures from experiments described in Fig 3B. The cell viabilities of Vero cells were assessed by Trypan Blue dye exclusion staining using a Countess II Automated Cell Counter. Each dot represents the cell viability from a single well +/- SEM counted in triplicates.

Multiple alphaviruses are known to infect macrophage cells, which are believed to play a role in alphavirus-induced arthritis [18, 34]. To assess the potential of scMAYV-E immune sera to protect against macrophage infection, an *in vitro* infection assay was performed. Addition of wild-type MAYV TRVL 15337 to human monocyte-derived macrophages (MDMs) progressively decreased the cell viability over 48 hours post infection (Fig 3A). Importantly, preincubation of MAYV TRVL 15337 with pooled sera from scMAYV-E immunized mice significantly increased the viability of MDMs whereas MDMs incubated with MAYV and pVax1 sera demonstrated high levels of cell death, which were observed in all visual fields using a phase-contrast and a fluorescent microscope (Fig 3B). Percent of live cells labeled with Labelling Dye Green seen as fluorescent green cells were evaluated by six independent reviewers (Fig 3C). These results led to the conclusion that while MAYV was likely inducing cell death, the sera from scMAYV-E immunized mice can significantly increase MDMs viability in the presence of MAYV. To further investigate this observation, we co-cultured for 36 hours Vero CCL-81 cells in a 6-well plate with supernatants from the infected MDMs preincubated with scMAYV-E sera or pVax1 sera. Vero cells maintained in the presence of supernatant from MDMs incubated with MAYV+scMAYV-E immune sera had a cell viability over 60% when compared to a 40% cell viability of Vero cell cultured in the presence of MDMs supernatant incubated with MAYV+pVax1 sera (Fig 3D). Taken together, these results suggest that scMAYV-E immune sera are capable of inhibiting viral replication in MDMs.

### scMAYV-E induces potent antigen-specific cellular immune responses

**Fig 4:**
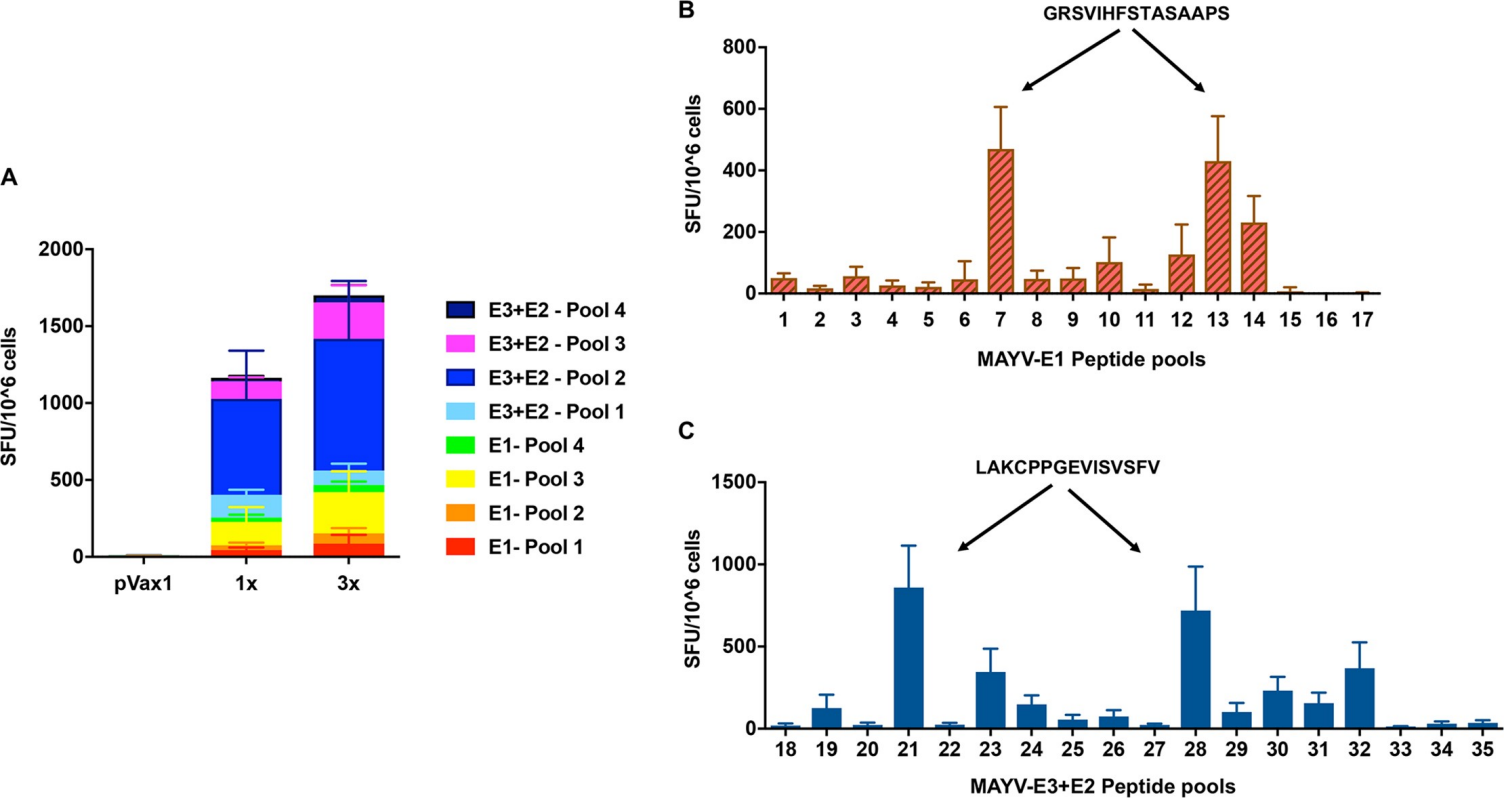
scMAYV-E induces a robust antigen specific cellular immune response to multiple epitopes in mice. C57BL/6 mice were immunized with 25 μg of either pVax1 empty vector or scMAYV-E plasmid once and euthanized 2 weeks later, or three times at 2-week intervals then euthanized one week after the last immunization. Splenocytes were harvested and cultured overnight in the presence of linear peptide pools spanning the full-length envelope protein. (A) IFN-γ ELISpot assay used to measure IFN-γ-producing spot-forming units (SFUs) generated per 10^6^ splenocytes +/- SEM. IFN-γ ELISpot assay performed on splenocytes from immunized animals after *ex vivo* stimulation with matrix peptide pools spanning the (B) E1 protein or (C) E3+E2 proteins. Average IFN-γ SFUs generated per 10^6^ splenocytes +/- SEM for each peptide pool shown. The immunodominant epitopes in E1 and E3+E2 identified via the matrix peptide pools are indicated with arrows.

We next evaluated anti-MAYV cellular immunity in splenocytes collected from the scMAYV-E immunized C57BL/6 mice. One week after the third immunization (day 35), pVax1 control and scMAYV-E immunized mice were euthanized, and bulk splenocytes were obtained for ELISpot assay. Briefly, harvested splenocytes from mice were *ex vivo* stimulated with various peptide pools encompassing the full-length MAYV envelope protein (i.e., glycoprotein E1, E2, and E3). The identity of each peptide pool is shown in Table 1. The antigen-specific production of interferon gamma (IFN-γ) by the cells is reported as spot forming units (SFUs) per million cells. Mice immunized (3x) with scMAYV-E exhibit a robust cellular response to multiple peptide pools throughout the MAYV envelope glycoprotein domains (Fig 4A). A similar, robust cellular response to multiple MAYV peptide pools was also observed in a separate cohort of C57BL/6 mice that received a single immunization of scMAYV-E vaccine (1x) (Fig 4A).

**Table 1:** Peptide pools encompassing the scMAYV-E sequence. 15-mer peptides overlapping by 9 amino acids spanning the entire length of the scMAYV-E sequence were created. 72 peptides comprise the E1 domain of scMAYV-E, and 81 peptides comprise the E3+E2 region. The E1 peptides were grouped into linear Pools 1–4 and E3+E2 into Pools 1–4, all of which consist of 20 or fewer peptides per pool. Matrix pools for E1 peptides and E3+E2 peptides were created separately.

scMAYV-E	Peptides included	E1	Peptides included	E3+E2	Peptides included
**E1- Pool 1**	1–20 (E1)	**Pool 1**	1–9 (E1)	**Pool 18**	1–9 (E3+E2)
**E1- Pool 2**	21-40(E1)	**Pool 2**	10-18(E1)	**Pool 19**	10-18(E3+E2)
**E1- Pool 3**	41-60(E1)	**Pool 3**	19-27(E1)	**Pool 20**	19-27(E3+E2)
**E1- Pool 4**	61-72(E1)	**Pool 4**	28-36(E1)	**Pool 21**	28-36(E3+E2)
**E3+E2- Pool 1**	1–20 (E3+E2)	**Pool 5**	37-45(E1)	**Pool 22**	37-45(E3+E2))
**E3+E2- Pool 2**	21–40 (E3+E2)	**Pool 6**	46-54(E1)	**Pool 23**	46-54(E3+E2)
**E3+E2- Pool 3**	41–60 (E3+E2)	**Pool 7**	55-63(E1)	**Pool 24**	55-63(E3+E2)
**E3+E2- Pool 4**	61–81 (E3+E2)	**Pool 8**	64-72(E1)	**Pool 25**	64-72(E3+E2)
		**Pool 9**	1,10,19,28,37,46,55,64(E1)	**Pool 26**	73-81(E3+E2)
		**Pool 10**	2,11,20,29,38,47,56,65(E1)	**Pool 27**	1,10,19,28,37,46,55,64,73(E3+E2)
		**Pool 11**	3,12,21,30,39,48,57,66(E1)	**Pool 28**	2,11,20,29,38,47,56,65,74(E3+E2)
		**Pool 12**	4,13,22,31,40,49,58,67(E1)	**Pool 29**	3,12,21,30,39,48,57,66,75(E3+E2)
		**Pool 13**	5,14,23,32,41,50,59,68(E1)	**Pool 30**	4,13,22,31,40,49,58,67,76(E3+E2)
		**Pool 14**	6,15,24,33,42,51,60,69(E1)	**Pool 31**	5,14,23,32,41,50,59,68,77(E3+E2)
		**Pool 15**	7,16,25,34,43,52,61,70(E1)	**Pool 32**	6,15,24,33,42,51,60,69,78(E3+E2)
		**Pool 16**	8,17,26,35,44,53,62,71(E1)	**Pool 33**	7,16,25,34,43,52,61,70,79(E3+E2)
		**Pool 17**	9,18,27,36,45,54,63,72(E1)	**Pool 34**	8,17,26,35,44,53,62,71,80(E3+E2)
				**Pool 35**	9,18,27,36,45,54,63,72,81(E3+E2)

To better define the dominant epitope(s) of scMAYV-E that elicit cellular responses in the C57BL/6 mouse model, an ELISpot mapping analysis was performed on bulk splenocytes from the mice that received three immunizations of scMAYV-E. Thirty-five matrix peptide pools encompassing the entire MAYV envelope protein, each comprised of individual 15-mer peptides that overlap by 9 amino acids, were created (Table 1) and *ex vivo* stimulated splenocytes for the IFN-γ ELISpot assay as previously described. Several matrix pools from different regions of the MAYV envelope glycoprotein domains were identified by SFU/10^6^ cells, with matrix pools 7 and 13 eliciting the highest responses in the E1 region as well as 21 and 28 in the E3+E2 regions (Fig 4B and 4C). Subsequent mapping analysis identified dominant epitopes within the MAYV E1 glycoprotein as ‘GRSVIHFSTASAAPS’ (Fig 4B) and within the MAYV-E3+E2 glycoproteins as ‘LAKCPPGEVISVSFV’ (Fig 4C). The amino acid sequences of the dominant epitopes determined from the ELISpot mapping analysis were confirmed using the immune epitope database analysis resources tools (http://tools.iedb.org), substantiating the effective antigen processing of scMAYV-E vaccine in this strain of mice.

### scMAYV-E generates significant polyfunctionality in both CD4^+^ and CD8^+^ T cells

**Fig 5:**
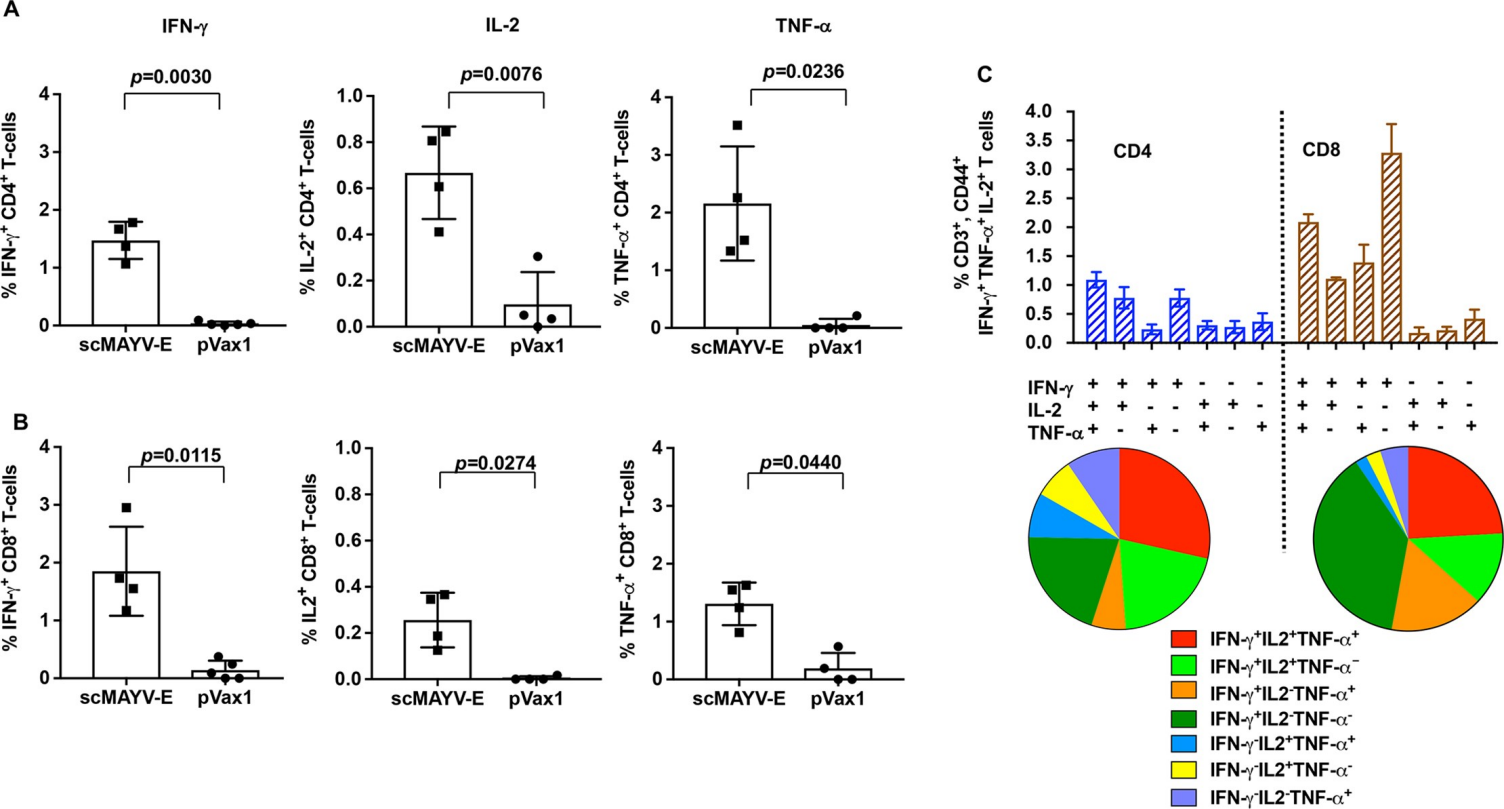
scMAYV-E induces both CD4+ and CD8+ T cell responses in mice. Splenocytes from immunized mice as outlined in the experiments from Fig 5 were also evaluated by polychromatic flow cytometry to identify the frequency of (A) CD4^+^ and (B) CD8^+^ T cells that produce the cytokines IFN-γ, IL-2, and TNF-α following a 5 hour *ex vivo* stimulation with pooled MAYV envelope peptides spanning the entire length of the envelope protein. One representative experiment of three is shown in A and B. (C) Frequency of total CD4^+^ and CD8^+^ T cells expressing each of the seven analyzed combinations of IFN-γ, TNF-α, and IL-2 using Boolean gating is shown as bar graphs. The pie charts represent the proportion of CD4+ and CD8+ T cells producing one, two, or all three cytokines.

To further characterize the cellular response induced by the scMAYV-E vaccine, splenocytes collected from the C57BL/6 mice receiving three immunizations of scMAYV-E or pVax1 control were evaluated by polychromatic flow cytometry. A panel of fluorophore-tagged antibodies was created and used to characterize helper (CD4^+^) and cytotoxic (CD8^+^) T cells production of the activated-state cytokines such as IFN-γ, tumor necrosis factor-α (TNF-α), and interleukin 2 (IL-2) post *ex vivo* stimulation of bulk splenocytes with peptides comprising MAYV full-length envelope. CD4^+^ and CD8^+^ T cells isolated only from scMAYV-E vaccinated mice were able to produce each intracellular cytokine upon stimulation with MAYV peptides (Fig 5A and 5B). scMAYV-E vaccination also induced polyfunctional responses in both T cell subsets (i.e., production of multiple activated-state cytokines from a single T cell) and indicated the presence of triple-positive T cells in both subsets (IFN-γ+IL2+TNF-α+% as red) (Fig 5C). Combined with the ELISpot results, scMAYV-E DNA vaccine induces both cellular immunity to MAYV and polyfunctionality of antigen-specific T cells.

### scMAYV-E-induced immunity protects mice from MAYV disease

We next evaluated whether the vaccine-induced MAYV-specific responses could protect against MAYV infection or disease in an animal challenge model. Previous studies showed that older immunocompetent mouse models do not exhibit arthritogenic signs of disease upon alphavirus challenge [29, 35]. We therefore chose to use the interferon α/β receptor knockout mouse (IFNAR^-/-^; A129) model, which has a defective innate immune response to pathogens. We established that a dose of 10^2^ PFU of MAYV administered i.p. produced measurable clinical signs of disease including weight loss, foot swelling, and criteria for euthanasia.

**Fig 6:**
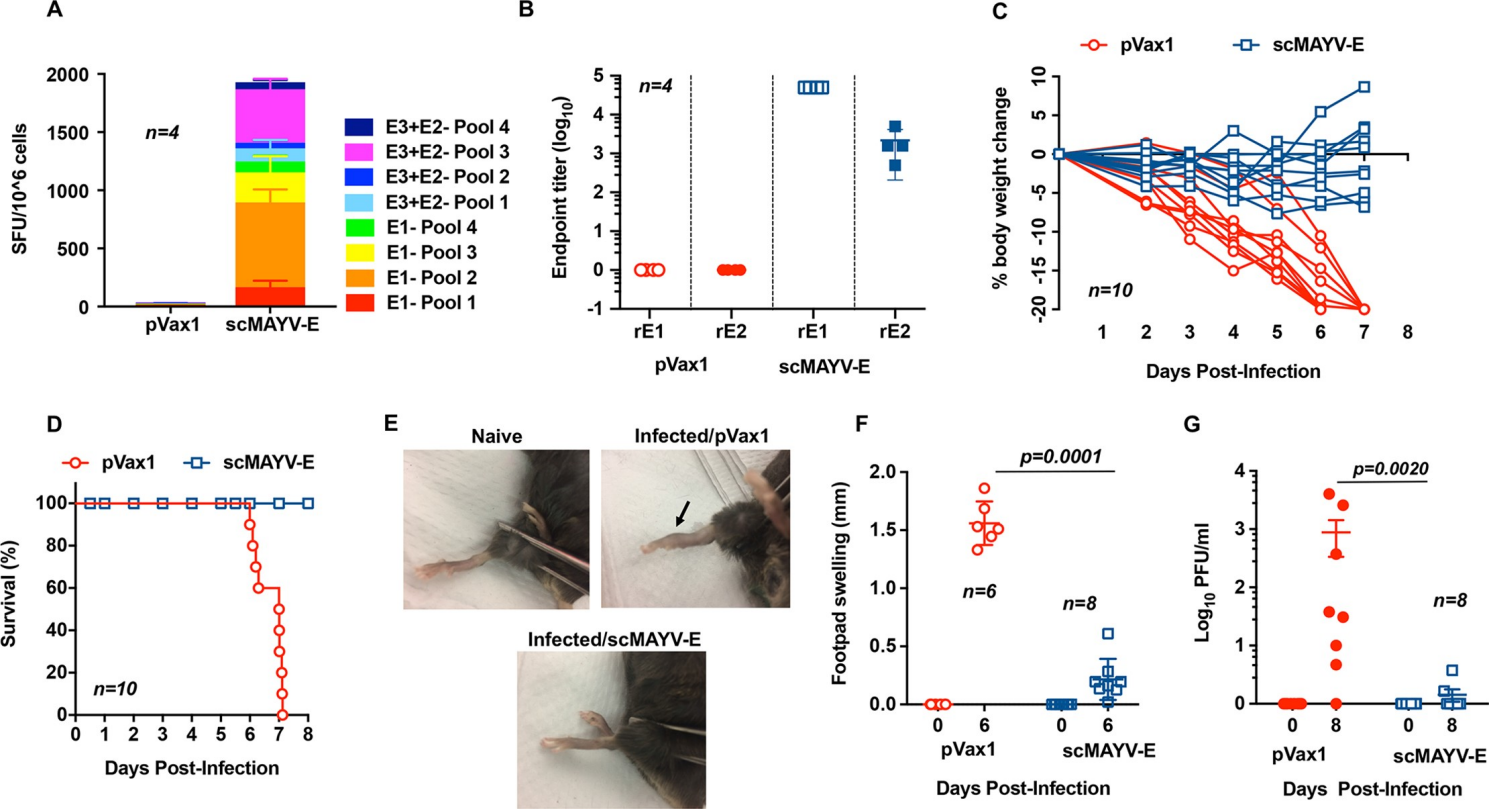
scMAYV-E protects immunized mice from MAYV challenge. IFNAR^-/-^ mice aged 4–6 weeks old were immunized twice, two weeks apart with pVax1 or scMAYV-E using EP-enhanced i.m. injection. Groups of mice for immunogenicity studies were euthanized one week after the final immunization. (A) Evaluation of cellular responses in vaccinated IFNAR^-/-^ mice. IFN-γ ELISpot of pVax1 or scMAYV-E immunized splenocytes is shown *(n = 4)*. (B) Evaluation of humoral responses in vaccinated IFNAR^-/-^ mice one week after the second immunization prior to viral challenge. Endpoint titers for rE1-IgG and rE2-IgG were evaluated for pVax1 or scMAYV-E immune sera *(n = 4)*. Mice were challenged intraperitoneally (i.p.) one week after the second immunization (day 21) with 10^2^ PFU of MAYV TRVL 15537. All mice were observed daily for clinical signs of disease up to 8 days post challenge. (C) Percent change in bodyweight from day 0 in individual immunized mice post challenge *(n = 10);* p = 0.0115 and (D) a Kaplan-Meier survival curve of scMAYV-E or pVax1 immunized mice post-MAYV challenge *(n = 10*; survival (%): scMAYV-E = 100, pVax1 = 0). (E) Representative pictures of rear footpad of uninfected mouse (naive), pVax1 immunized mouse (Infected/pVax1), and scMAYV-E immunized mouse (Infected/scMAYV-E) at 6 days post challenge. (F) Quantification of rear footpad size as measured by a caliper on day 6 post-MAYV challenge (*n = 6* pVax1*; n = 8* scMAYV-E). (G) MAYV PFU/ml in sera collected from pVax1 and scMAYV-E immunized mice at day 6 post MAYV challenge *(n = 8)*.

First, the cellular and humoral immunogenicity of scMAYV-E vaccinated IFNAR^-/-^ mice were evaluated as previously described with C67BL/6 mice to confirm that they mount similar adaptive immune responses. IFN-γ ELISpot responses (Fig 6A) and antigen-specific IgG endpoint titers (Fig 6B) were comparable to those observed in the C57BL/6 mice. For the challenge studies, cohorts of 10 four- to six-week old IFNAR^-/-^ mice were similarly immunized twice, two weeks apart, with either 25 μg scMAYV-E vaccine or pVax1 empty vector plasmid as a control. Animals were challenged on day 21, one week after the second immunization, with 10^2^ PFU of wild-type MAYV and were checked daily for 8 days for clinical signs of infection. All mice receiving pVax1 empty vector plasmid exhibited significant and progressive weight loss (Fig 6C). In contrast, mice vaccinated with scMAYV-E initially had minor weight loss over the first 4 days of challenge but exhibited slight weight gain after day 5 post challenge (Fig 6C). Percent body weight change on Day 7 comparing scMAYV-E and pVax1 groups had a p-value of 0.0115. Importantly, 100% of vaccinated mice survived the challenge, while all control mice met euthanasia criteria by 6–7 days post challenge (Fig 6D). pVax1 group also had significant footpad swelling at day 6 post challenge (Fig 6E and 6F). Quantification of MAYV in sera collected 6 days post challenge showed that scMAYV-E vaccinated mice had a significant reduction in circulating virus compared to pVax1 injected mice (Fig 6G). Combined, these data demonstrate that immune responses induced by the scMAYV-E vaccine provide protection from morbidity and viral load following MAYV challenge in this murine challenge model.

### scMAYV-E induced humoral responses are required to confer protection from viral challenge

**Fig 7:**
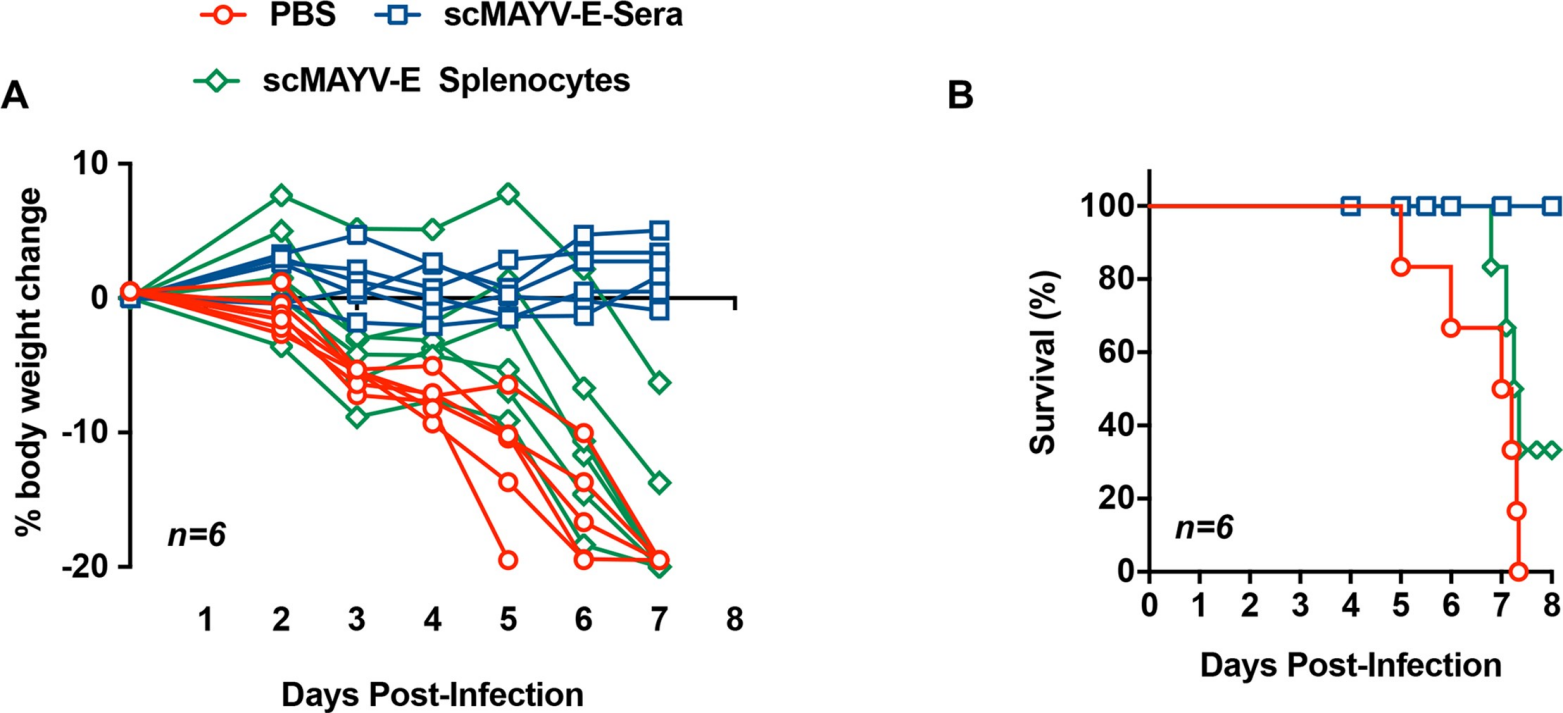
scMAYV-E induced humoral responses drive protection from MAYV challenge. IFNAR^-/-^ mice aged 4–6 weeks were immunized twice with 25 μg of scMAYV-E two weeks apart using EP-enhanced i.m. injection then euthanized one week after last immunization; immune sera and bulk splenocytes were collected. A naive batch of IFNAR^-/-^ mice of mixed sex aged 4–6 weeks were divided into three groups and injected i.p. with 1) 200 μl of immune sera from scMAYV-E immunized mice (blue), 2) 2x10^6^ bulk splenocytes from scMAYV-E immunized mice (green), or 3) PBS as a negative control (red). One hour post passive transfer, all mice were challenged with 10^2^ PFU of MAYV TRVL 15537 and observed daily for clinical signs of disease up to 8 days post challenge (*n = 6)*. (A) Percent change in bodyweight from day 0 shown for individual mice in each group; p = 0.0033 (B) A Kaplan-Meier survival curve shown for each group (*n = 6;* survival (%): scMAYV-E Sera = 100, scMAYV-E Splenocytes = 33.33, PBS = 0).

We next evaluated the relative contribution of the scMAYV-E vaccine induced humoral and cellular responses in an *in vivo* passive and adaptive transfers of MAYV challenge model. In this investigation, a cohort of 6 four- to six-week-old IFNAR^**-/-**^ mice were immunized twice at a two-week interval with 25 μg of scMAYV-E or pVax1. One week following the final immunization, the mice were euthanized and blood and bulk splenocytes were collected from each animal. Sera and splenocytes from each group was pooled. Cohorts of 6 naive, four- to six-week old IFNAR^**-/-**^ mice were injected with either PBS, 200 μl of pooled immune sera, or 2x10^6^ pooled splenocytes containing T cells, then subsequently challenged with 10^2^ PFU of MAYV i.p one hour post passive and adaptive transfers. Challenged mice were monitored for up to 8 days for clinical signs of disease. All mice receiving PBS prior to challenge progressively lost weight and were eventually euthanized due to severe disease as expected between day 5 and 7. Adoptive transfer of T cells from immunized mice provided some protection from weight loss (Fig 7A) and partial protection from disease (Fig 7B). Significantly, 100% of mice receiving scMAYV-E immune sera exhibited no weight loss (Fig 7A) and all survived the challenge (Fig 7B). Combined, these data establish the scMAYV-E induced humoral response as the main driver of its protective efficacy in this murine MAYV challenge model.

## Discussion

Mayaro virus is an emerging infectious disease agent endemic in tropical regions of South America, but recent evidence suggests that its range may be expanding into Central America and island nations of the Caribbean Sea [2, 11, 35]. The virus causes an acute febrile illness with symptoms including rash, headache, nausea, and diarrhea that can turn into a debilitating, long-term arthralgia in some patients after acute infection has cleared [36, 37]. There are currently no approved vaccines or therapeutics to combat MAYV disease and spread. Here, we report on the generation and immunogenicity of scMAYV-E, a synthetic, enhanced DNA vaccine encoding a novel consensus-designed sequence of the MAYV-envelope glycoproteins. Immunization of mice with scMAYV-E using enhanced EP delivery induced robust, MAYV-specific humoral and cellular responses. Importantly, these responses can neutralize MAYV infection *in vitro* and can fully protect susceptible mice from morbidity and mortality following MAYV challenge. The results show that scMAYV-E is a highly immunogenic vaccine candidate that warrants further testing in additional systems and animal models for developing countermeasures against MAYV infection and diseases.

The precise correlates of protection for MAYV have not been defined. A recent one-year longitudinal study of confirmed MAYV-infected individuals in Peru found that infection elicited robust anti-viral immune responses including strong neutralizing antibody responses and secretion of pro-inflammatory immune cytokines including IL-13, IL-7, and VEGF [28]. They also report that the strong neutralizing antibody response was not sufficient to prevent long-term negative outcomes of MAYV infection; however, these humoral responses developed post infection. Studies on related alphaviruses, including CHIKV, strongly suggest that a potent, neutralizing antibody response primarily mediates protection from infection, but non-neutralizing antibodies may contribute to protection as well through alternative effector functions [38]. Post infection, there is likely an important role for cellular immunity that may complement the humoral responses. Further studies will be needed to address this important issue.

The scMAYV-E DNA vaccine elicits both humoral and cellular responses against MAYV and consequently be an important tool to provide comprehensive protection from MAYV infection and disease. Antibodies to MAYV are generated after the initial priming immunization with scMAYV-E, and these responses increase after both one and two boosts in terms of binding capacity and affinity to rE1 and rE2. Immune sera from vaccinated mice was able to detect full-length MAYV envelope in scMAYV-E transfected cells as well as MAYV infected cells. scMAYV-E vaccination of mice was able to induce neutralizing antibodies that can block viral entry and inhibit cell death induced by MAYV infection in human MDMs. Passive transfer of immune sera from scMAYV-E vaccinated mice to susceptible naive IFNAR^-/-^ mice prior to MAYV challenge completely protected animals from illness, further confirming the importance of a strong humoral response for conferring protection from alphavirus infection.

Although the anti-MAYV T cell response appears less important for an immediate protection against MAYV infection, it may still be essential for the prevention of chronic disease by eliminating virus-infected cells. The cellular components induced by the scMAYV-E DNA vaccine target multiple epitopes along the full-length MAYV envelope glycoprotein. The strongest cellular responses were directed to epitopes in the E3+E2 domains of the envelope, whereas the responses to epitopes in the E1 glycoprotein were less robust. The epitope mapping studies using ELISpot assays identified two immunodominant epitopes, ‘LAKCPPGEVISVSFV’ in the E3+E2 domain and ‘GRSVIHFSTASAAPS’ within the E1 domain, providing important and useful reagents for studies of the T cell immune response in this haplotype. Interestingly, adaptive transfer of splenocytes from scMAYV-E immunized mice to susceptible naive IFNAR^-/-^ mice prior to MAYV challenge provided partial protection from weight loss and clinical symptoms of MAYV disease, suggesting that MAYV-specific cellular responses do contribute to protection. In this adoptive transfer experiment, MAYV-specific T cells were not purified or enriched from bulk splenocytes prior to transfer, thus it is possible that the partial protection observed here could be enhanced with a larger dose of antigen-specific T cells.

The immunogenicity of the scMAYV-E DNA vaccine mirrors what we observed in a previous DNA vaccine candidate targeting chikungunya virus (CHIKV-E) which encodes a synthetic consensus sequence of the full-length chikungunya envelope protein [27]. The CHIKV-E vaccine was similarly able to generate humoral and cellular responses directed towards the CHIKV envelope protein, and these responses could protect mice from morbidity and mortality following a CHIKV challenge [27].

The synthetic DNA vaccines have practical advantages for development including ease of manufacture and stability at warmer temperatures, likely reducing the requirement for a cold chain. They are non-live and non-replicating and do not integrate, thus providing conceptual safety advantages as well. Since DNA vaccine vectors do not induce anti-vector serology, they can be administered multiple times with no loss in potency and without interfering with other vaccine protocols. Such logistical and safety advantages warrant further studies of this vaccine approach, especially pertaining to diseases prevalent in resource-poor tropical settings where MAYV is the most prevalent. To the best of our knowledge, scMAYV-E is the third vaccine candidate for MAYV developed. The first vaccine was an inactivated Mayaro virus, and the second candidate reported was a live-attenuated MAYV vaccine [19, 20]. Both prior vaccines were shown to induce anti-MAYV humoral responses that could protect mice from MAYV challenge, but neither study reported on the induction of cellular responses to MAYV. The synthetic scMAYV-E DNA vaccine described here generates MAYV-specific humoral and cellular responses without viral replication, which is likely important for immune-challenged, young, pregnant, and elderly populations of potential travelers and residents in endemic areas in need of vaccine-induced immune protection. Additional studies of this vaccine approach using the DNA platform will provide further insight into the relative merits of such methods in the field.
